# MAVS disruption impairs downstream signaling and results in higher virus replication levels of salmonid alphavirus subtype 3 but not infectious pancreatic necrosis virus *in vitro*

**DOI:** 10.3389/fimmu.2024.1401086

**Published:** 2024-06-06

**Authors:** Cheng Xu, Amr A. A. Gamil, Xiaolin Wang, Hetron Mweemba Munang’andu, Øystein Evensen

**Affiliations:** ^1^Department of Paraclinical Sciences, Faculty of Veterinary Medicine, Norwegian University of Life Sciences, Ås, Norway; ^2^Fishteck AS, Oslo, Norway; ^3^Faculty of Biosciences and Aquaculture, Nord University, Bodø, Norway

**Keywords:** MAVS, Sensing, salmonid alphavirus, infectious pancreatic necrosis virus, disruption, TALEN

## Abstract

The mitochondrial anti-viral signaling (MAVS) protein is an intermediary adaptor protein of retinoic acid-inducible gene-1 (RIG-I) like receptor (RLR) signaling, which activates the transcription factor interferon (IFN) regulatory factor 3 (IRF3) and NF-kB to produce type I IFNs. MAVS expression has been reported in different fish species, but few studies have shown its functional role in anti-viral responses to fish viruses. In this study, we used the transcription activator-like effector nuclease (TALEN) as a gene editing tool to disrupt the function of MAVS in Chinook salmon (*Oncorhynchus tshawytscha*) embryonic cells (CHSE) to understand its role in induction of interferon I responses to infections with the (+) RNA virus salmonid alphavirus subtype 3 (SAV-3), and the dsRNA virus infectious pancreatic necrosis virus (IPNV) infection. A MAVS-disrupted CHSE clone with a 7-aa polypeptide (GVFVSRV) deletion mutation at the N-terminal of the CARD domain infected with SAV-3 resulted in significantly lower expression of IRF3, IFNa, and ISGs and increased viral titer (1.5 log_10_) compared to wild-type. In contrast, the IPNV titer in MAVS-disrupted cells was not different from the wild-type. Furthermore, overexpression of salmon MAVS in MAVS-disrupted CHSE cells rescued the impaired type I IFN-mediated anti-viral effect against SAV-3.

## 1 Introduction

Viral infection-elicited type I interferons (IFNs) production is crucial for host defense against viral infections (1). IFNs are induced following recognition through cell-surface or intracellular pattern recognition receptors (PRRs), which include toll-like receptors (TLRs) (2), the cytoplasmic retinoic acid-inducible gene-1 (RIG-I) like receptors (RLRs) (3), and stimulator of IFN genes (STING) protein (4), with RNA viruses being recognized by the two former. Once bound to viral RNA, TLRs signal via the adaptor MyD88 (5). In contrast, RLRs signal via the mitochondria-antiviral signaling (MAVS) protein (6) to activate the transcription factors IRF3/IRF7 and NF-κB, ultimately leading to type I IFN production. The MAVS protein contains an N-terminal caspase recruitment domain (CARD), a proline-rich (Pro) region (PRR), as well as a C-terminal mitochondrial transmembrane (TM) sequence (6), and the RLRs interact with MAVS protein at the mitochondrial membrane. After viral RNA binding, RLRs undergo conformational changes that expose and multimerize their CARD domains, which allows the CARD domains to interact with the N-terminal CARD of MAVS. The homotypic CARD-CARD associations relay the signal to TANK-binding kinase 1 (TBK1) and IκB kinase-ϵ (IKKϵ) (7), which in turn phosphorylate and activate interferon regulatory factor 3 (IRF3) and IRF7. Together with the transcription factors, nuclear factor-κB (NF-κB) translocates to the nucleus and induces the expression of type I interferons (3). Type I IFN activates the Janus kinase (JAK) signal transducer and activator of the transcription (STAT) pathway, which results in the expression of IFN-stimulated genes (ISGs) that control innate and adaptive immunity and diverse intracellular antimicrobial programs (8, 9).

The importance of the MAVS adaptor in RLR signaling pathways has been shown in knockout (KO) mice (10, 11) that have severe defects in RIG-I-mediated type I IFN responses (10), resulting in high susceptibility to RNA viral infections. MAVS KO-mice also fail to induce a type I IFN response to poly(I:C) stimulation (11) and have severely compromised immune defense against infection with vesicular stomatitis virus (VSV), an RNA virus of the Rhabdoviridae family.

Previous studies used a *de novo* transcriptome assembly to analyze the PRRs that recognize salmonid alphavirus subtype 3 (SAV-3) infection in a macrophage cell line (TO-cells) derived from Atlantic salmon leukocytes (12). We found that SAV-3 infections enriched pathways related to endosomal TLR (TLR3 and TLR8) and RLRs, of which the RLR signaling pathway was the most enriched (12). This suggested that the RLR pathway played a significant role in sensing SAV-3 infection, and it has been shown that teleost fish possess a functional RLR pathway in which MAVS may play a role in the induction of the innate immune response (13) where overexpression induces an anti-viral state *in vitro*. However, its native function in viral infections was not studied in detail. Farmed and wild teleost fish are infected with many RNA viruses where (+) RNA and dsRNA virus infections cause high losses, particularly in farmed salmon (14–17). Hence, understanding MAVS’ contribution to innate responses to SAV-3 (+RNA virus) and infectious pancreatic necrosis (IPN) virus (dsRNA) will shed light on crucial innate and adaptive defense mechanisms of salmonid teleost, and a recent study has shown that MAVS-knock out *in vitro* impairs PRR-signaling in fish cells (18).

We developed an MAVS-disrupted fish cell line using the transcription activator-like effector nucleases (TALEN) method (19, 20). We found that MAVS disruption lowers anti-viral responses, resulting in significantly higher replication levels of SAV-3, and the anti-viral response can be rescued by over-expressing MAVS in disrupted cell clones. In contrast, MAVS disruption has no impact on the replication of IPNV. This points to early-stage sensing of virus infection in fish cells being like what has been described for higher vertebrates, *i.e.*, the released viral RNA from SAV-3 carrying 5’-triphosphates following infection is sensed through RLRs in contrast to IPNV, which lacks 5’-triphosphates. Disrupting the core part of the CARD domain impairs the downstream axis and results in higher progeny production (1.5 log). In contrast, it is assumed that anti-viral responses to IPNV are not elicited through RLR sensing since RNA synthesis is intraparticle (mRNA and genome), which shields viral sensing, and any released dsRNA at later stages lacks 5’-triphosphates. These findings have implications for anti-viral strategies in disease control for farmed fish, including approaches to imprint or direct adaptive immune responses for optimal vaccine design.

## 2 Materials and methods

### 2.1 Construction of TALEN

A pair of primers, MAVS-F1 and MAVS-R1 (**Table 1**), were designed to amplify partial 5’ mRNA and genomic DNA sequence of the MAVS gene of CHSE cells by RT-PCR and PCR, respectively, based on the MAVS mRNA sequence of Atlantic salmon (GenBank accession no: XM_014130694). The PCR amplicons were cloned into the pGEM-T Easy vector (Promega) for sequencing. The MAVS exon 1/intron 1 structure was determined by aligning the mRNA to the genomic DNA sequence. The complete exon-intron structure of Chinook salmon MAVS (GenBank accession number: NC_056436) was derived by automated computational analysis using the gene prediction method Gnomon (21). The CARD domain, the proline-rich region (PRR), and the transmembrane (TM) domain were determined by protein sequence alignment of Chinook salmon MAVS to human MAVS (GenBank accession number: NP_065797). The appropriate TALEN targeting site was designed using the online TAL Effector Nucleotide Targeter 2.0 software program (https://tale-nt.cac.cornell.edu/node/add/talen-old). To establish the MAVS-disrupted clones, a pair of TALEN expression vectors targeting MAVS exon 1 that encode the N-terminal of the CARD domain was constructed using the FastTALE™ TALEN Assembly Kit (SiDanSai Biotechnology Co., Ltd, China). The target sequences of the MAVS TALENs were as follows: left 5’-TGCACCTGCGGCGCAGA-3’ and right 5’-CTGTGGCTTTCACTCTG-3’. The left arm TALEN vector contained the Puromycin resistance gene, while the right arm TALEN vector contained the EGFP, which was co-expressed with TALEN and suited for selecting transfected cells.

**Table 1:** Primer sequences used for PCR and qPCR.

Primer name	Sequence	Use	GenBank Accession no
MAVS-F1	ATGTCGTCGTTCACCCGTGAA	Exon1 cloning PCR identification	XM_014130694
MAVS-R1 MAVS-R2	GATTCAGTTTGGGACAGGGCTAGGTGTG CTGGTTGTGCTTGTTCCACT		
IRF3-F	TGGACCAATCAGGAGCGAAC	qPCR	FJ517643
IRF3-R	AGCCCACGCCTTGAAAATAA		
IFNa-F	TGGGAGGAGATATCACAAAGC	qPCR	AY216594
IFNa-R	TCCCAGGTGACAGATTTCAT		
Mx-F	TGCAACCACAGAGGCTTTGAA	qPCR	U66475
Mx-R	GGCTTGGTCAGGATGCCTAAT		
IFIT5-F	GCTGGGAAGAAGCTTAAGCAGAT	qPCR	BT046021
IFIT5-R	TCAGAGGCCTCGCCAACT		
β-actin-F	CCAGTCCTGCTCACTGAGGC	qPCR	AF012125
β-actin-R	GGTCTCAAACATGATCTGGGTCA		

### 2.2 Cell culture, electroporation, and sorting

Chinook salmon embryo 214 (CHSE-214) cells were transfected by electroporation with 20 µg paired MAVS TALEN plasmids (each 10 µg) per 10^6^ cells using the Neon transfection system (Invitrogen, Carlsbad, CA) with one pulse of 1200V for 40 ms. The transfected cells were cultured in Leibovitz’s L-15 (L15) medium supplemented with 10% fetal bovine serum (FBS) at 20˚C against the selection of puromycin (Invitrogen) at 2.5 μg/ml. After three days, the surviving cells were collected by trypsinization, subjected to a second electroporation under the same conditions, and cultured with puromycin for another three days. After that, about 10^6^ cells were pooled and subjected to fluorescence-activated cell sorting (FACS) based on the green fluorescent-marker expression by FACS Aria II cell sorter (BD Biosciences). After sorting, the cells were put in one well of a 24-well plate to allow for recovery in the L15 growth medium.

### 2.3 Establishment of MAVS-disrupted clones

After FACS, the cells were allowed to recover for 10 days, after which single colonies were isolated by limiting dilution and replated individually to wells of 96-well plates. Single colonies were allowed to grow to near confluence, and then they were split to replicate wells of a 24-well plate to create a working and frozen stock. The working stock was cultured to confluence, and genomic DNA was extracted using the DNeasy Blood & Tissue Kit (Qiagen, Hilden, Germany). In contrast, the frozen stock was stored in liquid nitrogen. Genotyping at the TALEN target site was then performed for each clone by PCR amplification (94°C, 30 s; 55°C, 30 s; 68°C, 45 s) using AccuPrime™ Taq DNA Polymerase (Invitrogen, Paisley, UK) and a primer pair (MAVS-F1 and MAVS-R2) designed to yield amplicons containing the target site (**Table 1**). Amplicons were subjected to electrophoresis on 1% agarose gels to discriminate clones with indels, with positive clones having a band that shifted in size. PCR amplicons were cloned into the pGEM-T Easy vector (Promega, Madison, WI, USA) and transformed into *E. coli* TOP10 competent cells using the heat shock method for later isolation of subsets of potentially positive clones. The successful transformation was confirmed by doing colony PCR of selected colonies. Plasmids were extracted from the cultures with positive colony PCR results using the QIAprep Spin Miniprep Kit (Qiagen, Hilden, Germany). They were sent to Eurofins Genomics, Germany, for Sanger sequencing to confirm the presence of mutant alleles. CHSE clones with confirmed mutant alleles were retrieved from the frozen stocks and expanded for further experiments.

To confirm the MAVS gene disruption at mRNA level, we isolated total RNA from the identified positive clones using the RNeasy mini Kit (Qiagen, Hilden, Germany) with on-column DNase treatment according to the manufacturer’s instructions, and 1 μg of total RNA was subjected to cDNA synthesis using the SuperScript III reverse transcriptase system (Invitrogen, Paisley, UK) and oligo(dT)20 primers in a total volume of 20 μL. The synthesized cDNA was subjected to PCR amplification, cloning to pGEM-T Easy vector, and sequencing analysis as described above.

### 2.4 Effect of MAVS disruption on mRNA expression of type I IFN-related genes after SAV-3 infection

MAVS-disrupted clone 20 (Clone 20) and wild-type CHSE cells (CHSE_WT_) were grown in 6-well plates to 80% confluent. After infection with 1 MOI SAV-3 in triplicates, the cells were incubated at 15°C in L15 media with 2% FBS. Total RNA from infected cells was collected after 48 h and used to analyze mRNA expression of type I IFN-related genes using quantitative RT-PCR (qRT-PCR) as previously described (22). Primer sequences used for qRT-PCR are shown in **Table 1**. The 2 ^-ΔΔCT^ method (23) was used to quantify the fold change in gene expression levels in MAVS-disrupted clone 20 and CHSE_WT_ cells after SAV-3 infection relative to the non-infected CHSE_WT_ cells. All quantifications were normalized using the β-actin reference gene.

### 2.5 Effect of type I IFN treatment on ISG expression and SAV-3 replication in MAVS-disrupted clone 20

The recombinant salmon type I IFN (IFNa1) used in this study was made in our laboratory, as previously described by Xu et al. (24). Recombinant salmon IFN-I was 10-fold serially diluted from 0.47 mg/ml and incubated with MAVS-disrupted clone 20 and CHSE_WT_ cells. QRT-PCR measured induction of Mx and IFIT5 expression at 24 h post-treatment of IFN-I. Cells were infected with 1 MOI of SAV-3 after treatment of IFN-I for 24 h. Total RNA from infected cells was collected three days post-infection, and qRT-PCR determined intracellular virus replication levels. The data were expressed as the mean fold changes in gene expression of different dilutions of an IFN-I-treated group relative to the non-treated CHSE_WT_ control group after normalization to β-actin. Inhibition of SAV-3 replication by IFN-I was expressed as the mean fold changes in E2 gene expression of different dilutions of an IFN-I-treated group relative to the non-treated, infected CHSE_WT_.

### 2.6 Effect of MAVS disruption on SAV-3 and IPNV replication *in vitro*

MAVS-disrupted and CHSE_WT_ cells were separately propagated in an L15 medium supplemented with 10% FBS and were incubated at 20°C. The SAV-3 (Genbank accession JQ799139) and a recombinant IPN virus (rNVI-15Rb) used to inoculate the cells have previously been described (14, 22, 25). MAVS-disrupted clones 5, 20, and CHSE_WT_ cells were grown in 6-well plates to 80% confluent and infected with 1 MOI SAV-3 and 10 MOI IPNV in triplicates, respectively, followed by incubation at 15°C in L15 medium with 2% FBS. Culture supernatants from IPNV-infected cells were collected at seven days, and from SAV-3 infected cells were collected ten days post-infection, respectively. The virus was titrated in CHSE-214 cells using the 50% tissue culture infective dose (TCID_50_) method (26).

### 2.7 Effect of MAVS overexpression on SAV-3 replication in MAVS-disrupted clone 20

Eukaryotic expression plasmid pcDNA-MAVS expressing the salmon MAVS gene was a kind gift from Dr. Pierre Boudinot (13). MAVS-disrupted clone 20 was transfected with 2 µg (per 2x10^6^ cells) of pcDNA-MAVS or empty vector pcDNA3.1 using the Neon transfection system with 2 pulses of 1200V for 40 ms. Four days post-transfection, cells were infected with SAV-3 at an MOI 1. MAVS-disrupted clone 20 without transfection and CHSE_WT_ cells were also infected with SAV-3 at an MOI 1 and served as controls. The culture supernatants were collected 5- and 10 days post-infection, and the viral titers were determined using the TCID_50_ method in CHSE cells.

### 2.8 Statistical analysis

Differences in virus titers and gene expression levels were determined by Student’s t-test and One- or Two-way ANOVA (as specified) using GraphPad Prism version 10.0.0 for Mac, GraphPad Software, Boston, Massachusetts USA, www.graphpad.com. The significant level for rejection of Ho was set at p < 0.05.

## 3 Results

### 3.1 TALEN-mediated disruption of MAVS in CHSE cells

**Figure 1:**
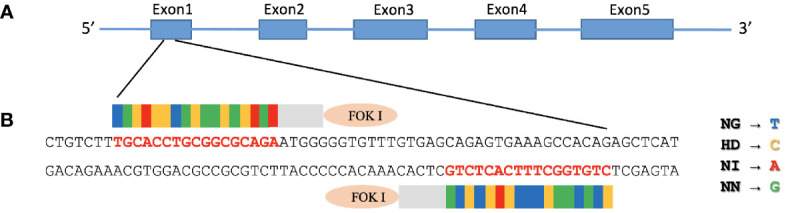
Construction of TALENs targeting MAVS gene. **(A)** Diagram illustrating the structure of Chinook salmon MAVS (GenBank accession number: NC_056436). MAVS contains three domains: an N-terminal caspase recruitment domain (CARD), a middle proline-rich region (PRR), and a C-terminal transmembrane (TM) domain. **(B)** FastTALE™ TALEN Assembly Kit was used to construct TALENs targeting MAVS gene, exon 1 of salmon MAVS gene, that encode N-terminal of CARD domain was targeted by left and right TALEN arms with a 17-bp spacer. The TALEN-targeting nucleotides are shown in bold red letters. The repeat variable di-residues (RVDs) in TAL effectors are NG, HD, NI, and NN, which specify the nucleotides T, C, A, and G, respectively.

We started by aligning the MAVS 5’ mRNA with the genomic DNA sequence, giving CHSE exon 1 a size of 123 bp from the ATG start codon (**Figure 1A**). CHSE cells were transfected with the TALEN pair that targets the exon 1 region of the MAVS gene (**Figure 1B**), 29 bp downstream of the initiator ATG codon.

**Figure 2:**
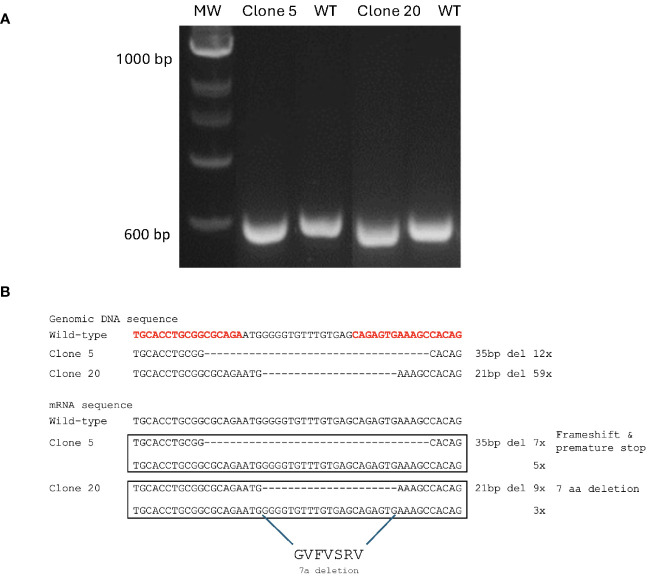
Generation of MAVS-disrupted clones in CHSE cells. **(A)** Agarose gel electrophoresis of PCR amplicons after amplification of genomic DNA sequence containing the TALEN targeting site of MAVS gene in clones 5, 20, and CHSE_WT_ cells. **(B)** Genomic DNA and mRNA sequence of the TALEN targeting site in each allele of MAVS-disrupted clones 5 and 20, compared with CHSE_WT_ cells. A deletion of 35 bp was found in one allele of clone 5, and a deletion of 21 bp in one allele of clone 20, and the corresponding amino acid sequence is shown. The TALEN-targeting nucleotides are shown in bold red letters.

The right arm TALEN vector expressed EGFP that suited the observation of successful transfection, and the GFP-positive cells were enriched with a FACS Aria II cell sorter after two rounds of transfection followed by puromycin selection. After sorting, these cells were plated to recover in an L15 growth medium for ten days, and single-cell clones were then obtained by limiting dilution. Genomic DNA was prepared from 30 single clones where the region containing the TALEN targeting site of the MAVS gene was amplified by PCR and analyzed by electrophoresis. The primer set (MAVS-F1 and MAVS-R2) yielded a 612 bp amplicon for the CHSE_WT_ cells. In contrast, clones 5 and 20 yielded amplicons of slightly smaller size (**Figure 2A**). These amplicons were cloned into the pGEM-T Easy vector and confirmed by sequencing where all 12 reads of clone 5 contained a deletion of 35 base pairs in the MAVS exon 1, that caused a frameshift and premature stop. All 59 reads of clone 20 contained a deletion of 21 base pairs that caused 7-aa polypeptide (GVFVSRV) deletion mutation (**Figure 2B**), corresponding to amino acids 18-24 of MAVS protein, located at the N-terminal of the CARD domain (**Figure 1A**).

To confirm the gene deletion in the MAVS gene at the mRNA level, cDNA was synthesized from the total RNA of clones 5 and 20, and the region containing the TALEN targeting site of the MAVS gene was amplified by PCR using primer set (MAVS-F1 and MAVS-R2), that yielded a 297 bp amplicon for the CHSE_WT_ cells (results not shown). The amplicons from clones 5 and 20 were cloned into the pGEM-T Easy vector and sequenced, revealing that reads containing a deletion mutation of MAVS in mRNA sequence for both clones 5 and 20 differed from what was found for the genomic DNA sequencing. For clone 5, seven of 12 reads contained the deletion of 35 base pairs in the MAVS exon 1. The remaining 5 clones were equal to the wild-type (WT). For clone 20, 9 of 12 reads contained the deletion of 21 base pairs. In comparison, 3 of 12 reads were equal to WT (**Figure 2B**). Considering the whole genome duplication in salmon, these results indicate that 2/4 MAVS alleles of clone 5 contained the deletion mutation that caused a frameshift and premature stop. In contrast, 3/4 MAVS alleles of clone 20 had deletion mutation that resulted in a shortened protein with a deletion of 7-aa polypeptide (GVFVSRV) at the N-terminal of the CARD domain.

### 3.2 MAVS disruption increased SAV-3 but not IPNV replication

**Figure 3:**
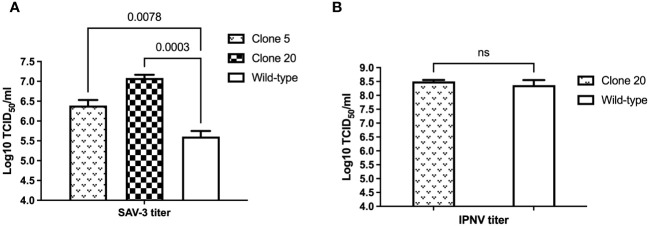
Effect of MAVS disruption on **(A)** SAV-3 and **(B)** IPNV replication *in vitro*. MAVS-disrupted clone 5, 20, and wild-type CHSE cells were grown in 6-well plates and infected with 1 MOI SAV-3 or 10 MOI IPNV in triplicates, respectively. SAV-3 replication levels at 10 dpi and IPNV replication levels at 7 dpi were measured by titration of the virus in culture supernatants using the TCID_50_ method. Representative data from three independent experiments are shown (mean ± SEM, n = 3). p-values are indicated for wild-type vs. clone 5 and clone 20 for SAV3 by One-way ANOVA and Dunnet’s *post-hoc* test, and Student’s t-test for IPNV. ns, not statistically significant (p>0.05).

In light of clones 5 and 20 are composed of cells of different proportions of alleles of the MAVS gene being disrupted, this allowed us to perform a functional, semiquantitative study, and both cell clones were infected with SAV-3 or IPNV and compared to CHSE_WT_. Infections were done at MOI of 1 for SAV-3 and 10 for IPNV, respectively. MAVS-disrupted clones 5 and 20 both gave a significant increase in virus titers for SAV-3, with less effect for clone 5 with a lower proportion of disrupted alleles, *i.e.*, 0.8 log_10_ (p<0.05) TCID_50_/mL increase in virus titers for clone 5 and 1.5 log_10_ (p<0.001) for clone 20, compared to CHSE_WT_ 10 days post-infection (**Figure 3A**). MAVS-disrupted clone 20 showed earlier and more widespread CPE than CHSE_WT_ cells after infection of SAV-3 (results not shown). For IPNV-infected cells, there was no difference in endpoint virus titer for MAVS-disrupted clone 20 and CHSE_WT_ cells at seven days post-infection (p>0.05, **Figure 3B**).

Since higher editing efficiency and SAV-3 replication levels were observed in clone 20, we used this clone for further functional experiments.

### 3.3 MAVS disruption impaired the expression of type I IFN-related genes after SAV-3 infection

**Figure 4:**
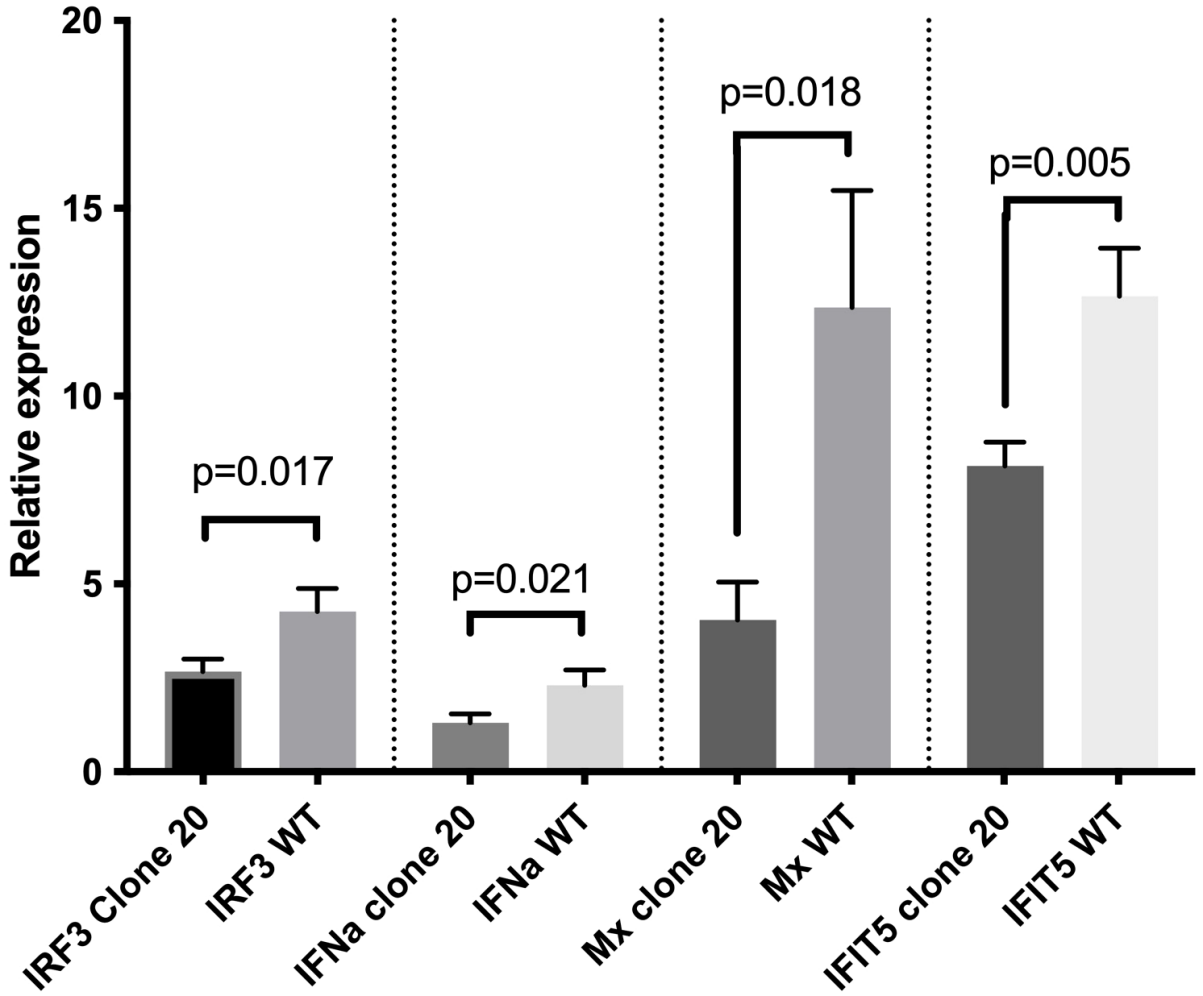
Effect of MAVS disruption on mRNA expression of type I IFN-related genes after SAV-3 infection. qRT-PCR measured IRF3, IFNa, Mx, and IFIT5 mRNA expression levels at 2 days post SAV-3 infection in MAVS-disrupted clone 20 and wild-type CHSE cells. Representative data from two independent experiments are shown (mean ± SEM, n = 3 per experiment), and statistical comparisons are made using Student’s t-test.

We anticipated that the 7-aa polypeptide (GVFVSRV) deletion mutation at the N-terminal of the CARD domain led to functional disruption of MAVS in clone 20 and next examined if this clone had impaired downstream responses compared to CHSE_WT_. To test this, we first measured mRNA expression levels by qRT-PCR of interferon regulatory factor 3 (IRF3), and then IFNa, Mx, and IFN-induced protein with tetratricopeptide repeats 5 (IFIT5) after SAV-3 infection. We found that CHSE_WT_ had significantly higher mRNA expression levels (p<0.05) of IRF3, IFNa, Mx, and IFIT5 compared with MAVS-disrupted clone 20 at two days post SAV-3 infection (**Figure 4**). mRNA expression for IRF3, IFNa, Mx and IFIT5 were 4.3-, 2.3-, 12.4- and 12.7-fold upregulated in CHSE_WT_ at two days post SAV-3 infection (compared to non-infected CHSE_WT_), while 2.7-, 1.3-, 4.0- and 8.1-fold upregulated in MAVS-disrupted clone 20 (**Figure 4**). From this we concluded that the 7-aa polypeptide (GVFVSRV) deletion at the N-terminal of the CARD domain disrupted MAVS function, impaired the sensing process and ultimately expression of type I IFN induced genes downstream of MAVS in the RLR signaling pathway allowing for higher virus replication levels/production of progeny.

**Figure 5:**
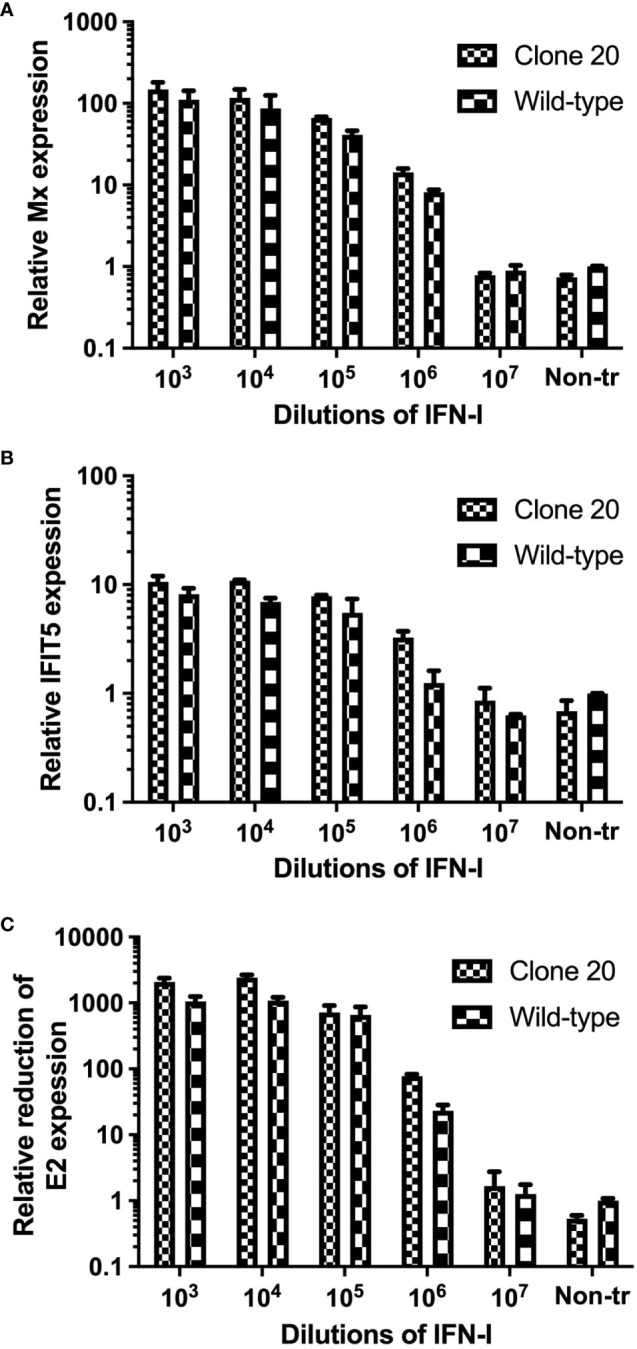
Effect of type I IFN treatment on ISGs expression and SAV-3 replication in MAVS-disrupted clone 20. mRNA expression quantified by qRT-PCR of ISGs for **(A)** Mx and **(B)** IFIT5, and **(C)** reduction of SAV-3 E2 expression after treatment with recombinant salmon IFN-I at different concentrations. MAVS-disrupted clone 20 was compared to CHSE_WT_ cells. Data expressed as mean ± SD (n=2).

MAVS disruption did not affect ISG expression levels and anti-SAV-3 capacity when clone 20 cells were treated with type I IFN. Next, we needed to ensure that the observed effect in MAVS-disrupted CHSE clone 20 was explicitly due to the impairment of MAVS signaling and unrelated to type I IFN-mediated JAK-STAT signaling. First, we treated clone 20 cells with different dilutions of recombinant salmon IFN-I (rIFN-I) (24) and assayed the induction of different ISGs by qRT-PCR. No significant difference in mRNA expression levels of anti-viral ISGs (Mx and IFIT5) (p>0.05) was observed between MAVS-disrupted clone 20 and CHSE_WT_ treated with different concentrations of IFN-I (**Figures 5A, B**). In addition, no significant difference (p>0.05) in inhibition of SAV-3 replication by rIFN-I was observed between MAVS-disrupted clone 20 and CHSE_WT_ after treatment with different concentrations of IFN-I (**Figure 5C**). This allowed us to conclude that MAVS disruption did not compromise the ability of the cells to mount effective antiviral responses induced through type I IFN-mediated JAK-STAT signaling.

### 3.4 MAVS overexpression rescued impaired anti-viral effect against SAV-3 in MAVS-disrupted clone 20

**Figure 6:**
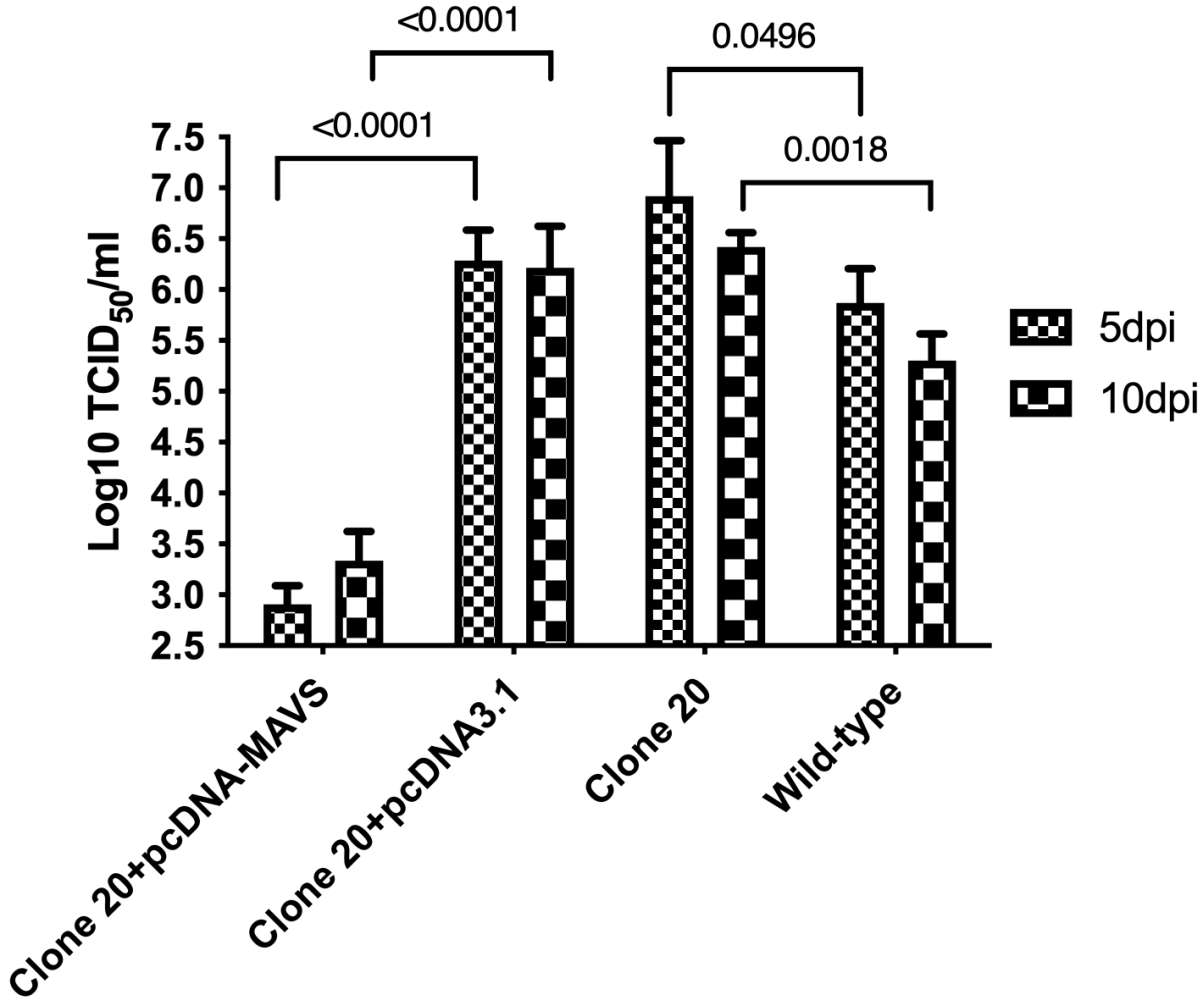
Effect of MAVS overexpression on SAV-3 replication in MAVS-disrupted clone 20. MAVS-disrupted clone 20 was transfected with pcDNA-MAVS or pcDNA3.1 empty plasmid. Four days post-transfection, cells were infected with SAV-3 at an MOI 1. MAVS-disrupted clone 20 without transfection and wild-type CHSE cells were also infected with 1 MOI SAV-3 and served as controls. SAV-3 replication levels at 5 and 10 dpi were measured by titration of the virus using the TCID_50_ method. Data expressed as mean ± SEM (n=3), using 2-way ANOVA and Tukey’s multiple comparison test.

Then, we explored if the anti-viral abilities of clone 20 could be rescued by transfection with an MAVS-expressing plasmid (knock-in effect). Salmon MAVS protein was overexpressed by transfecting clone 20 cells with a pcDNA-MAVS plasmid, where controls included clone 20 transfected with an empty plasmid. Clone 20 cells transfected with the MAVS-expressing plasmid had a significant decrease (p<0.001) of 3.4 and 2.9 log_10_ TCID_50_/mL SAV-3 titers compared to clone 20 transfected with pcDNA3.1 empty plasmid at 5 and 10 dpi, respectively (**Figure 6**). Furthermore, MAVS-disrupted clone 20 without transfection and infected with SAV-3 had a significant increase in titers at 5 (p<0.05) and ten dpi (p<0.01) corresponding to a log increase of 1.05 and 1.12 log_10_ TCID_50_/mL compared with CHSE_WT_ (**Figure 6**).

## 4 Discussion

This study showed that MAVS disruption enhanced SAV3 replication, and MAVS-disrupted cells had significantly lower induction of IFNa after SAV-3 infection. Response to rIFN-I treatment was equal or better compared to CHSE_WT_ cells. Together, these findings provide good documentation that RLR-mediated signaling through MAVS is a crucial pathway for SAV-3 viral RNA sensing and type I IFN induction, playing an essential role in mediating protection against fish alphaviruses infection similar to what has been observed in mammalian alphavirus infections (27). Further, in line with early-stage genome synthesis, including mRNA transcription and early genome replication inside the particle, MAVS plays less of a role in eliciting anti-viral responses against dsRNA viruses. Hence, our findings support and extend previous reports (13) that the alphavirus sensing mechanism is conserved across vertebrate species through the RLR signaling pathway. MAVS has been implicated in sensing and response to RNA virus infections in different fish species, including zebrafish (*Danio rerio*) (28), Japanese flounder (*Paralichthys olivaceus*) (29), Sea perch (*Lateolabrax japonicas*) (30), and Atlantic salmon (*Salmo salar*, L.) (13). In Atlantic salmon, MAVS overexpressing cells produced high levels of IFNa and ISGs, resulting in a 10^4^-fold decrease in RNA virus replication (13). Salmon IFNa was mainly induced through the RLR pathway, where MAVS plays a crucial role in signal transduction (31), whereas IFNb and IFNc were the main IFNs induced through the TLR7 pathway (32). For other fish species, in Japanese flounder, overexpressing MAVS in cell culture led to upregulation of Mx, ISG15, and IRF3 linked to high protection against hirame rhabdovirus (HIRRV) and viral hemorrhagic septicemia virus (VHSV) infection. Cells overexpressing MAVS showed reduced CPE and low HIRRV and VHSV viral titers (29).

Similarly, the MAVS gene from Sea perch (*Lateolabrax japonicas*) was cloned, and overexpression induced by poly I:C resulted in decreased replication of nervous necrosis virus (NNV) concomitantly with high expression of IRF3, Mx, and ISG15. These studies show that MAVS is expressed in different fish species, and overexpression increases type I IFN and ISGs, ultimately suppressing RNA virus replication in fish cells (30). These studies are all based on overexpression of MAVS, but the underlying mechanisms have yet to be studied. In mammals, MAVS knockout (KO) mice and cells have been widely used to demonstrate the importance of this gene in the induction of type I IFN responses and its role in innate immunity. For example, studies of MAVS KO mice found that Dengue virus, a positive-sense, single-stranded RNA virus sensed by RIG-I and MDA5, replicated to higher titer in serum and lymphoid tissues. In contrast, IRF3 and type I IFN levels were low (33). Human metapneumovirus (hMPV), a negative-sense, single-stranded RNA virus sensed by RLR, produced high viral titer in MAVS KO mice concomitant with impaired type I IFN responses, contrasted with WT-mice with low viral titer and high type I IFN levels (34).

For mammalian alphavirus like Sindbis virus (SINV), it has been shown that its genomic and subgenomic RNA contain triphosphate at their 5’ ends (35), a molecular structure recognized explicitly by RIG-I. The presence of RIG-I and MDA5 determines the activation of the anti-viral response by SINV-infected cells. RIG-I or MDA5 is sufficient for sensing SINV viral RNA and induction of type I IFN, while no type I IFN response is induced without RIG-I and MDA5 (27). In previous studies, we showed that the RLR signaling pathway was the most significantly enriched pathway induced by SAV-3 infection in salmon cells (12), suggesting salmonid alphavirus is mainly sensed by RIG-I and MDA5 via recognition of its 5’-ppp-RNA and long dsRNA, respectively, which mediate induction of type I IFN and anti-viral ISGs in salmon cells. This study shows that MAVS disruption reduced mRNA expression levels of different type I IFN-related genes, including IRF3, IFNa, Mx, and IFIT5 in clone 20.

In contrast, their expression levels were high in wild-type CHSE cells after SAV-3 infection. As a consequence, higher replication levels of SAV-3 (1.5 log_10_ increase in titer) and more severe CPE were observed in the MAVS-disrupted clone 20, indicating impaired type I IFN responses and weakened anti-viral protection against SAV-3 in salmon cells due to MAVS disruption. Combining the rescue or knock-in experiment in which MAVS overexpression in clone 20 inhibited SAV-3 replication levels with 2.9 log_10_ reduction in viral titer, these findings provide strong evidence that RLR signaling via MAVS is the critical pathway for type I IFN induction and innate anti-viral protection against SAV-3 at salmon cells.

However, not all RNA viruses are affected by MAVS knockout in their ability to induce type I IFN and anti-viral responses. Wu et al. showed that MAVS KO mice exhibited equivalent anti-viral and inflammatory gene responses, including type I IFN responses in lung tissues compared with WT-mice following influenza A virus (IAV) infection. MAVS is dispensable for survival in IAV infection *in vivo* (36), as Matsumoto et al. (37) recent evidence suggests that compartmentalization of the receptors and their adaptor molecule is vital for discrimination between self and non-self and for distinct innate immune signals. For example, RLRs are cytosolic sensors that recognize viral 5’-ppp-RNA and long dsRNA intracellularly, and they use the MAVS adaptor to induce type I IFN. At the same time, TLR3 is a transmembrane receptor that recognizes viral dsRNA in the endosomal compartment and uses the adaptor molecule TRIF to induce type I IFN (38–41). In fish, apart from TLR3, PKR can also play a role in sensing the intracellular viral dsRNA. We have previously studied the interplay between IPNV and PKR and showed that the virus exploits this anti-viral sensor and uses it to its advantage (42).

On the other hand, the extracellular viral dsRNA is sensed by TLR22 and has been identified in species including fugu (*Takifugu rubripes*) (43), turbot (*Scophthalmus maximus*) (44), and Atlantic salmon (45). At the same time, its signaling pathway has been detailed in Fugu. The fugu TLR22, localized at the cell surface, preferentially recognizes long dsRNA and can induce type I IFN after stimulation of IPNV genomic dsRNA (43). It is likely that IPNV, being a dsRNA virus lacking the 5’-ppp-RNA structure, is recognized by TLR3, TLR22, and PKR rather than RIG-I-like receptors. Therefore, its ability to replicate at the same level in the MAVS-disrupted clone 20 and CHSE_WT_ cells is not surprising and further suggests a minimal role of the RLR-mediated pathway through MAVS adaptor in inducing type I IFN during IPNV infection. However, there is a need for detailed investigations to elucidate the exact mechanisms used by IPNV to enter salmon cells and to explain the mechanisms it uses to induce type I IFN responses in infected cells.

We used TALEN as a gene editing tool to disrupt the function of MAVS in CHSE-214 cells. Multiple gene copies due to genome duplication pose a significant challenge in gene editing studies in salmon/salmon-derived cells (46). However, we managed to attain about 75% gene editing efficiency in one cell clone, which enabled us to perform functional studies using two different salmonid viruses, a single-stranded, positive-strand RNA virus (SAV-3) and a double-stranded RNA virus (IPNV) that are sensed at an early stage of infection through different pathways while IPNV replication remained unaffected.

In summary, this study has shown that MAVS has a significant role in the induction of type I interferon responses in CHSE cells infected by SAV-3. MAVS disruption results in reduced expression of IRF3, IFNa, and ISGs, resulting in increased levels of SAV-3 replication and more severe CPE in CHSE cells. Overexpression of MAVS rescued the impaired type I IFN-mediated anti-viral effect against SAV-3 in MAVS-disrupted CHSE cells. In addition, we have shown that TALEN can disrupt targeted gene function in salmon cells. As one of the earliest studies to show that the gene-editing tool TALEN can be used to introduce targeted gene mutation in fish cells, we advocate that the TALEN technique should be applied further in elucidating the functional mechanisms of signaling pathways in fish cells to enhance our understanding of fish immunology.

## Data availability statement

The original contributions presented in the study are included in the article/supplementary materials. Further inquiries can be directed to the corresponding author.

## Author contributions

CX: Conceptualization, Formal analysis, Methodology, Writing – original draft, Writing – review & editing. AG: Data curation, Formal analysis, Methodology, Writing – review & editing. XW: Conceptualization, Methodology, Writing – review & editing. HM: Formal analysis, Supervision, Writing – review & editing. ØE: Conceptualization, Data curation, Formal analysis, Funding acquisition, Project administration, Supervision, Writing – original draft, Writing – review & editing.
