# Corrigendum: MAVS disruption impairs downstream signaling and results in higher virus replication levels of salmonid alphavirus subtype 3 but not infectious pancreatic necrosis virus *in vitro*

**DOI:** 10.3389/fimmu.2024.1500204

**Published:** 2024-10-02

**Authors:** Cheng Xu, Amr A. A. Gamil, Xiaolin Wang, Hetron Mweemba Munang’andu, Øystein Evensen

**Affiliations:** ^1^ Department of Paraclinical Sciences, Faculty of Veterinary Medicine, Norwegian University of Life Sciences, Ås, Norway; ^2^ Fishteck AS, Oslo, Norway; ^3^ Faculty of Biosciences and Aquaculture, Nord University, Bodø, Norway

**Keywords:** MAVS, Sensing, salmonid alphavirus, infectious pancreatic necrosis virus, disruption, TALEN

In the published article, there was an error in affiliation 2. Instead of “Next Generation Sequencing (NGS) Oncology for Nordic & Baltic Region, Thermo Fisher Scientific, Oslo, Norway”, it should be “Fishteck AS, Oslo, Norway”.

In the published article, the **Conflict of Interest** section has been updated to include the new commercial affiliation for author XW. The correct **Conflict of Interest** statement appears below.

“Author XW was employed by company Fishteck AS.

The remaining authors declare that the research was conducted in the absence of any commercial or financial relationships that could be construed as a potential conflict of interest.

The author(s) declared that they were an editorial board member of Frontiers, at the time of submission. This had no impact on the peer review process and the final decision.”

The authors apologize for this error and state that this does not change the scientific conclusions of the article in any way. The original article has been updated.

